# Assessment of four sterilization techniques for meniscal allograft transplantation in rabbits

**DOI:** 10.1002/jeo2.70150

**Published:** 2025-01-20

**Authors:** X. S. Wang, H. G. Jia, D. Q. Gu, D. Z. Luo, Y. T. Zhao, Z. J. Liu, Y. D. Zhang

**Affiliations:** ^1^ Department of Orthopedics Children's Hospital Affiliated to Capital Institute of Pediatrics Beijing China; ^2^ Senior Department of Orthopedics the Fourth Medical Center of PLA General Hospital Beijing China; ^3^ Department of Orthopedics, Senior Department of Orthopedics the Fifth Medical Center of PLA General Hospital Beijing China

**Keywords:** biomechanics, meniscal allograft transplantation, morphological structure, sterilization

## Abstract

**Purpose:**

This study aimed to compare the effects of four sterilization techniques on meniscal allograft transplantation (MAT) in rabbits.

**Methods:**

In total, 85 medial or lateral meniscuses were obtained from 22 adult New Zealand white rabbits. These 85 meniscal allografts were seeded with *Staphylococcus aureus* (*S. aureus*) and randomly divided into five groups (*n *= 17): iodine group, Cobalt‐60 group, glutaraldehyde group, ethylene oxide group and control group. Bacterial colonies of the allografts were determined before (*n* = 7) and after (*n* = 7) sterilization. Histological features were analyzed using haematoxylin and eosin (H&E) staining (*n* = 3). An additional 50 medial or lateral meniscuses were taken from 13 adult New Zealand white rabbits, and they were kept fresh‐frozen (−40°C) for 1 month. Subsequently, these 50 meniscuses were randomly divided into five groups, which were treated with iodine, Cobalt‐60, glutaraldehyde, ethylene oxide and normal saline, separately (*n* = 10), and stored at −20°C before MAT. MAT was given to the knee joints of the right hind legs of 50 adult Japanese white rabbits. After 6 weeks of MAT, the transplanted meniscal allografts were taken for biomechanical test (*n* = 7) and H&E staining (*n* = 3).

**Results:**

The results of the bacterial count indicated that the number of *S. aureus* colonies was less than 1 colony‐forming unit (CFU)/allograft in all five groups after sterilization, except for the control group (415 CFU/allograft). H&E staining revealed that allografts in the iodine group presented the complete structure; allografts in the Cobalt‐60 and glutaraldehyde groups were preserved relatively well; and allografts in the control and ethylene oxide groups were severely destructed, especially in the ethylene oxide group. Using the transplanted allografts, the biomechanical test showed that the maximum load of allografts in each group was significantly different, with ethylene oxide effective sterilization agents being used for disinfecting meniscal grafts (iodine group: 270.71 ± 62.81 N, Cobalt‐60 group: 182.14 ± 71.29 N, glutaraldehyde group: 254.29 ± 31.55 N, ethylene oxide group: 40.00 ± 22.73 N and control group: 183.14 ± 76.40) (*p* ≤ 0.001). H&E staining with transplanted allografts revealed that allografts in the iodine group had the most intact structure; allografts in the Cobalt‐60 and glutaraldehyde groups presented a relatively intact structure; and allografts in the control and ethylene oxide groups were seriously damaged, especially in the ethylene oxide group.

**Conclusion:**

It was found that iodine, Cobalt‐60, glutaraldehyde and ethylene oxide are effective sterilization agents for disinfecting meniscal grafts. Iodine exhibited superior preservation of both the biomechanics and structural integrity of the meniscal allografts, whereas ethylene oxide caused the most severe destruction to the meniscal allografts.

**Level of Evidence:**

Not applicable because this is an animal study.

**Abbreviations**
CFU: colony‐forming units
H&E: haematoxylin and eosin
MAT: meniscal allograft transplantation
*S. aureus*
: *Staphylococcus aureus*

## INTRODUCTION

The meniscuses function as buffering absorbers that could decrease the contact area of the tibiofemoral joint surface, thereby stabilizing the knee joints [23]. Meniscal injuries would affect the kinematics of the knee, increase the peak contact pressure, and even facilitate the degenerative process of the knee joints [22]. The management of meniscal injuries includes conservative physical therapy combined with anti‐inflammatory medications or surgical intervention [26]. However, previous studies have demonstrated that meniscus resection could significantly increase the stress on the tibiofemoral joint surface and even lead to secondary osteoarthritis [3, 20, 27, 28]. Therefore, allogeneic meniscal allograft transplantations (MATs) promote restoring the native state of the biomechanics in the injured knee and provide effective treatment options for patients with meniscus injuries [31].

The first successful MAT was performed by Milachowski in 1984 [13]. MAT was initially regarded as an experimental attempt or salvage procedure to delay arthroplasty in patients with meniscectomy‐related arthritis [30]. After years of refinement in recipient indications, graft processing and preservation and operational skills, MAT has been proven effective in reducing pain and restoring knee joint function [30]. MAT has become an advantageous alternative intervention strategy for patients with meniscal injuries. Although MAT is relatively rarely conducted, which is estimated to be 1/1,000,000 population, the advantage of MAT is still striking it may improve the stability of the knee over meniscectomy [23, 25]. The disparities in the application of MAT are summarized as follows: (1) the long‐term benefit in preventing future osteoarthritis remains elusive; (2) economic and ethical issues; (3) the development of meniscal allograft preparation techniques and (4) surgical challenges [14].

Sterilization, defined as the procedure of eliminating all organisms, is critical in the preparation of allografts because it can prevent the transmission of bacteria or viruses from the donor to the recipient [33]. The risk of transmitting diseases through connective tissue allografts, including menisci, has been extensively documented [5]. Although the incidence of allograft‐derived infection is as low as 0.00015%, the transmission of pathogens (e.g., human immunodeficiency virus or hepatitis C virus) is still a health‐related concern [5]. Irradiation and chemical sterilization are common sterilization techniques for musculoskeletal allografts [7]. However, potential damages in the mechanical and biochemical properties of the allografts during the sterilization processes have remarkably attracted researchers' attention [33]. Numerous sterilization methods are inappropriate for meniscal allografts due to their adverse effects on grafts or recipients [5]. It has been proven that Gamma irradiation is associated with adverse effects on the biomechanical properties of meniscal allografts, while ethylene oxide is linked to inflammatory responses [9]. Therefore, the exploration of effective and safe sterilization techniques for MAT is essential.

The present study aimed to compare the long‐term efficacy of four common sterilization techniques for MAT in rabbits, including iodine, Cobalt‐60, glutaraldehyde and ethylene oxide.

## MATERIALS AND METHODS

### Study design

With the approval of the Institutional Animal Care and Use Committee of the Beijing Keyu (KY20230618005), all experimental procedures were conducted according to the guidelines for the laboratory animals.

This study was divided into two parts. In the first part, the effectiveness of four sterilization techniques on meniscuses was examined. *Staphylococcus aureus* (*S. aureus*) was selected for this experiment due to its clinical relevance, as it is a common pathogen responsible for joint infections, particularly in orthopaedic surgeries and allograft procedures. This choice aimed to replicate a real‐world clinical scenario, in which *S. aureus* contamination is a significant concern. While spores of Bacillus or yeasts are typically used for industrial sterilization quality control, the concentration of this study was to test sterilization effectiveness against a pathogen more likely to be encountered in clinical settings. The *S. aureus* strain used in this study was a commercial strain obtained from the American Type Culture Collection, ensuring consistency and reliability across experiments.

Dissection was performed on 85 medial or lateral menisci from 22 adult New Zealand white rabbits, each with a body weight of 2.5 ± 0.5 kg. These 85 meniscuses were randomly divided into five groups (*n* = 17): iodine group, Cobalt‐60 group, glutaraldehyde group, ethylene oxide group and control group. This indicated that each group contained 17 menisci. All the meniscuses were seeded with 10^6^ colony‐forming units (CFU)/mL *S. aureus* and incubated at 37°C for 48 h. From each group, seven meniscuses were randomly selected for microbial analysis (*n* = 7), and the number of *S. aureus* colonies was counted. Subsequently, four experimental groups of meniscal allografts were sterilized with iodine, Cobalt‐60, glutaraldehyde, and ethylene oxide, while the control group was treated with normal saline. The remaining meniscuses in each group were used for histological evaluation (*n* = 3) or reserved for further experiments.

In the second part of the experiment, the long‐term effects of four sterilization technologies on meniscal allografts were evaluated. Another 50 medial or lateral meniscuses were taken from 13 adult New Zealand white rabbits and kept fresh‐frozen (−40°C) for 1 month. Subsequently, all 50 meniscuses were randomly divided into five groups, treated with iodine, Cobalt‐60, glutaraldehyde, ethylene oxide or normal saline (*n* = 10). MATs were randomly implanted into the right knee joints of 50 Japanese white rabbits using these meniscal allografts. After 6 weeks, the transplanted meniscal allografts were retrieved for biomechanical testing (*n* = 7) and histological evaluation (*n* = 3).

Notably, the limit of detection for the bacterial colony counts was 1 × 10² CFU/allograft. CFU counts below this threshold were recorded as ‘<1 CFU/allograft’ in Table 1.

**Table 1:** Bacterial colony count of *S. aureus* before and after sterilization (*n* = 7).

Group	Before sterilization (CFU/allograft)	After sterilization (CFU/allograft)	Sterilization rate (%)
Iodine	629 ± 225.00	<1	100
Cobalt‐60	526 ± 79.00	<1	100
Glutaraldehyde	537 ± 251.00	<1	100
Ethylene oxide	600 ± 86.00	<1	100
Control	620 ± 202.00	415.00 ± 21.50	81.854
*p* Value	0.755	≤0.001	≤0.001

Abbreviations: CFU, colony‐forming unit; *S. aureus*, *Staphylococcus aureus*.

### Harvesting of the allogenic meniscuses

The New Zealand white rabbits, which were donors of meniscal allografts, were given euthanasia. Their medial or lateral meniscuses were then excised. The meniscuses were washed with normal saline to remove the residual blood and cut into sections with the appropriate shape.

### Bacterial colony count

The meniscuses used as samples for the bacterial colony count were placed in Eppendorf tubes and quickly frozen using liquid nitrogen. The frozen meniscuses were then crushed into powder and suspended in 1000 μL of normal saline. To ensure complete bacterial recovery, the samples were vortexed thoroughly in saline solution to detach the bacteria. The entire suspension was serially diluted before plating the dilutions on trypticase‐soy‐blood agar 3000*g* for 10 min to separate bacterial cells, and the supernatant was collected and plated. The plates were incubated at 37°C for 24 h to allow visible *S. aureus* colony formation. After the incubation period, the CFU was counted to determine the bacterial load.

### Sterilization of the meniscal allografts

In this study, five groups of menisci underwent different sterilization treatments. The menisci in the iodine group were immersed in a 10% iodine solution for 6 h to ensure adequate microbial inactivation. The Cobalt‐60 group was exposed to gamma radiation at a dose of 25 kGy for 24 h to achieve sterilization. The glutaraldehyde group was treated with a 2.5% glutaraldehyde solution for 6 h, while the ethylene oxide group was exposed to ethylene oxide gas for 24 h under controlled conditions. The control group was treated with normal saline for 6 h without any sterilization. Following the sterilization procedures, all menisci were thoroughly rinsed by immersing them three times in sterile normal saline for 6 h to remove any residual sterilizing agent.

### Surgical procedure

Meniscal allografts were immersed in a solution containing 80 mg of gentamicin (1.6 mg/mL) in 50 mL of normal saline and then trimmed to the appropriate size. The recipient Japanese white rabbits were anaesthetised with ketamine and sumantin. The skin of the transplant site was disinfected three times with iodine, and a medial parapatellar incision was made. A total meniscectomy was conducted by removing the medial meniscus of the right hind legs of the recipient rabbits. The size‐matched meniscal allograft was placed and sutured to the right hind knee cavity of the recipient. Subsequently, the medial collateral ligament, subcutaneous area and skin were sutured respectively.

### Haematoxylin and eosin (H&E) staining

The meniscus tissue was fixed in 4% paraformaldehyde for 24 h, followed by dehydration in graded ethanol, clearing in xylene and embedding in paraffin. The embedded meniscus was sectioned into 5‐µm‐thick slices using a microtome. H&E staining was performed to visualize the histological structure of the meniscus.

### Biomechanical test

The biomechanical test was performed using a computer‐controlled test machine (Instron) as previously described [13]. The meniscus was cut into 3 × 3 × 1 mm^3^ pieces. The working temperature was kept at 23°C, and the meniscus was hydrated with normal saline. The biomechanical test of the meniscus was performed according to the manufacturer's instructions.

### Statistical analysis

The quantitative data were analyzed using SPSS 16.0 software (IBM). The differences between the groups were examined via the Student–Newman–Keuls method and *q* test, and differences among the groups were analyzed by one‐way analysis of variance (ANOVA). *p* ≤ 0.05 was considered statistically significant.

## RESULTS

### Effectiveness of the four sterilization technologies on meniscus

The effectiveness of the four sterilization technologies on the meniscus was compared with the bacterial colony count. As presented in Table 1, the difference in *S. aureus* colony count of the meniscus before sterilization was not statistically significant among the five groups (*p* = 0.755), indicating that the bacterial burden of the meniscus was comparable across the five groups.

Following the adjustments in the bacterial retrieval method, including vortexing the samples and serially diluting the suspensions, as well as extending the incubation time to 24 h at 37°C, the CFU was counted. This allowed for a more accurate and reliable determination of the bacterial load across all groups. After sterilization, the bacterial colony count of *S. aureus* in the iodine, Cobalt‐60, glutaraldehyde and ethylene oxide groups was less than 1 CFU/allograft, while it was 415.00 ± 21.50 CFU/allograft in the control group (*p* < 0.001). The sterilization rate in the iodine, Cobalt‐60, glutaraldehyde and ethylene oxide groups was 100%, while the bacterial reduction rate in the control group was 81.85% (*p* < 0.001).

These results indicate that iodine, Cobalt‐60, glutaraldehyde and ethylene oxide were all potent disinfectants for meniscus sterilization, effectively reducing the bacterial load to near zero.

### Effects of the four sterilization technologies on the histological features of the fresh meniscuses

**Figure 1:**
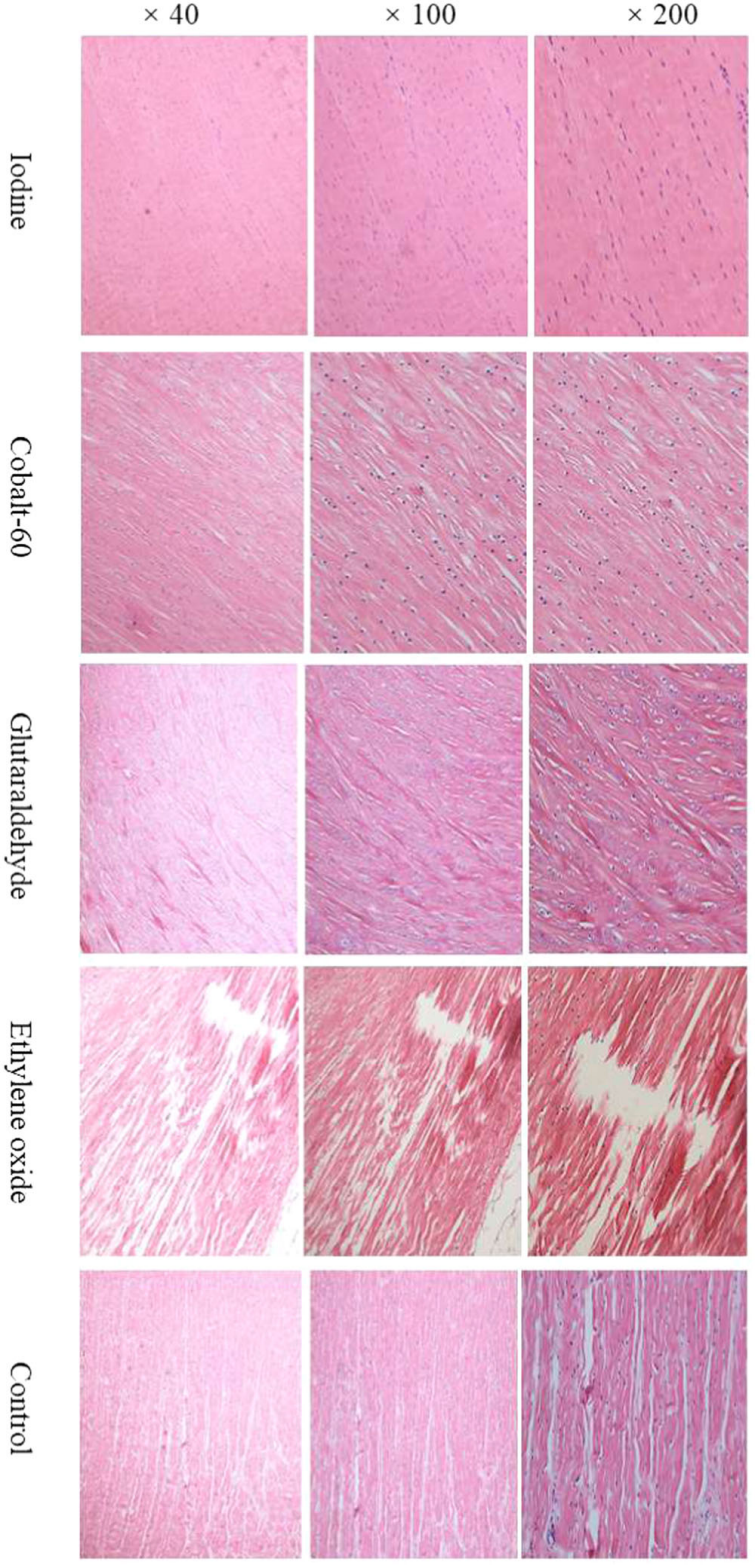
Histological features of the fresh meniscuses after sterilization.

After the sterilization process, the sampling meniscuses from five groups (*n* = 3) were stained with H&E, and their histological features were analyzed under a light microscope. As illustrated in Figure 1, the structures of the meniscuses in the iodine group were the most intact among the five groups, except for a very small number of uniformly distributed small pores within the meniscuses. The structures of the meniscuses in the Cobalt‐60 group structure were relatively well preserved, accompanied by widely distributed slender spindle‐shaped small pores, which were arranged in a regular and orderly manner. The structures of the meniscuses in the glutaraldehyde group were relatively well preserved, accompanied by widely distributed short spindle‐shaped small pores that were arranged irregularly. The structures of the meniscuses in the ethylene oxide group were the most severely damaged, which presented a disordered, tortuous and irregular arrangement, accompanied by widely distributed large cracks and regional tissue debris. The structural damage of the meniscuses in the control group was relatively severe, accompanied by regional tortuous and irregular arrangement and widely distributed tortuous cracks.

### Effects of the four sterilization technologies on the biomechanics of meniscal allografts after 6 weeks of transplantation

A biomechanical test was employed to evaluate the mechanical properties of the meniscal grafts after MAT. As presented in Table 2, the iodine group could tolerate the highest average load (270.71 ± 62.81 N), while the ethylene oxide group only withstood the lowest average load of (40.00 ± 22.73 N). The average maximal load in the Cobalt‐60, glutaraldehyde and control groups was 182.14 ± 71.29, 254.29 ± 31.55 and 183.14 ± 76.40 N, respectively.

**Table 2:** Biomechanics of the meniscal allografts after 6 weeks of transplantation (*n* = 7).

Group	Load (N)
Iodine	270.71 ± 62.81
Cobalt‐60	182.14 ± 71.29
Glutaraldehyde	254.29 ± 31.55
Ethylene oxide	40.00 ± 22.73
Control	183.14 ± 76.40
*p* Value	<0.000

**Figure 2:**
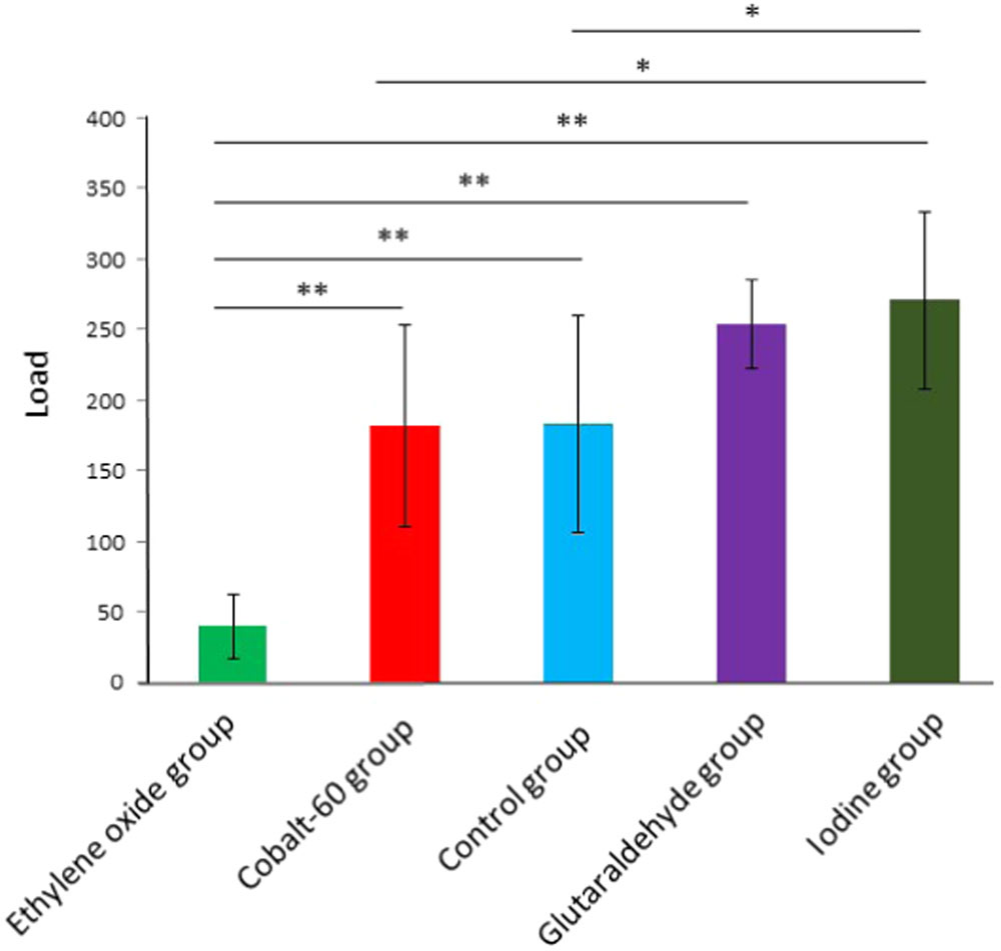
Biomechanics of the meniscal allograft after 6 weeks of transplantation. *p < 0.05 and **p < 0.01.

The statistical differences among the groups were evaluated using one‐way ANOVA (Figure 2). The results of pairwise comparison showed that the maximum load in the ethylene oxide group was significantly lower than in the Cobalt‐60, control, glutaraldehyde and iodine groups (*p* < 0.01). The maximum load in the iodine group was significantly higher than in the Cobalt‐60 and control groups (*p* < 0.05).

**Figure 3:**
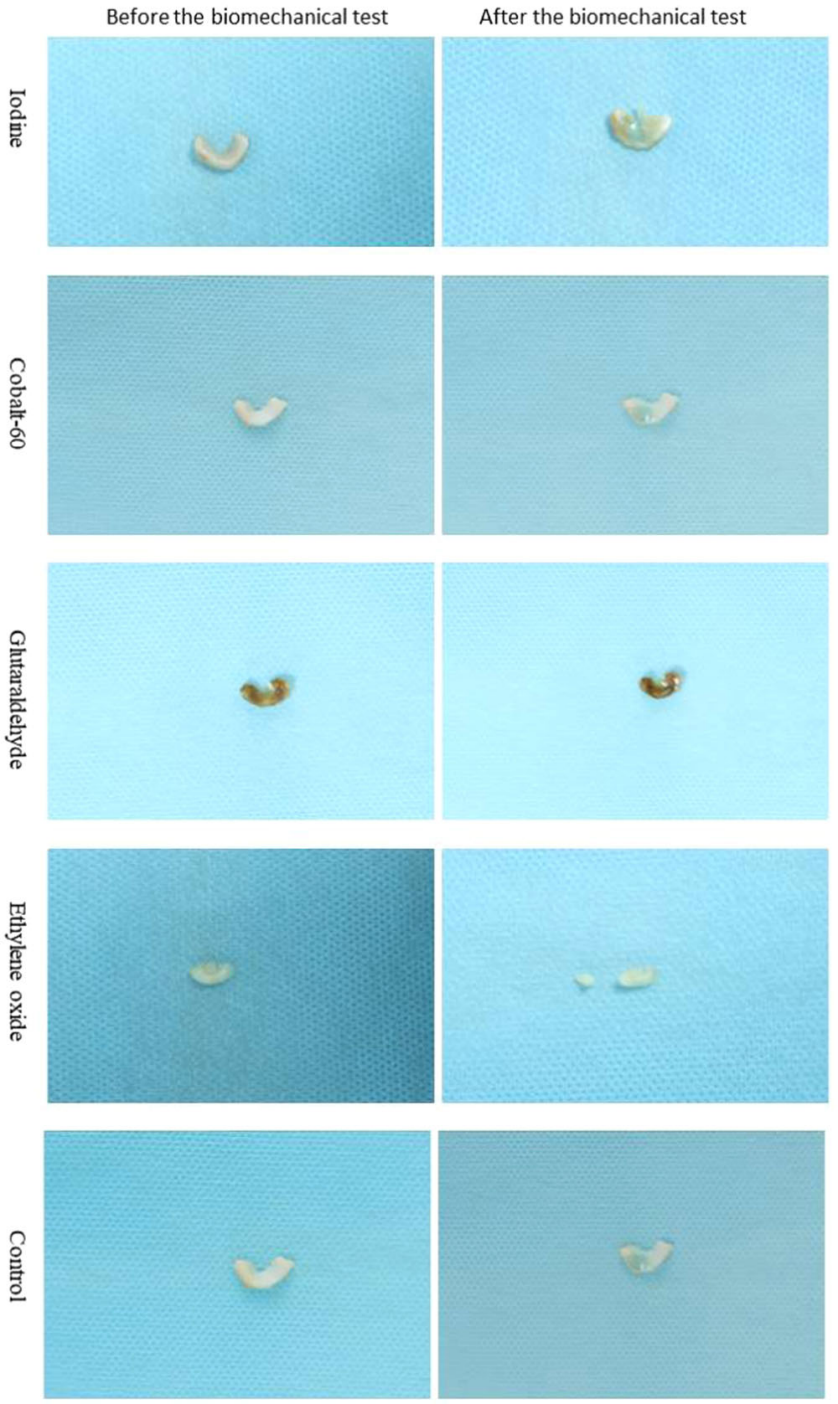
Representative graphs of the gross morphology of the meniscal allografts before and after the biomechanical test.

Figure 3 displays the representative gross morphology of the meniscal allografts before and after the biomechanical test. It was found that meniscal allografts in the ethylene oxide group were crushed into pieces after the biomechanical test. The morphology of meniscal allografts in the iodine, glutaraldehyde, control and Cobalt‐60 groups was less deformed. It was revealed that meniscuses in the glutaraldehyde group became dark after transplantation.

### Effects of the four sterilization technologies on the histologic features of meniscal allografts after 6 weeks of transplantation

**Figure 4:**
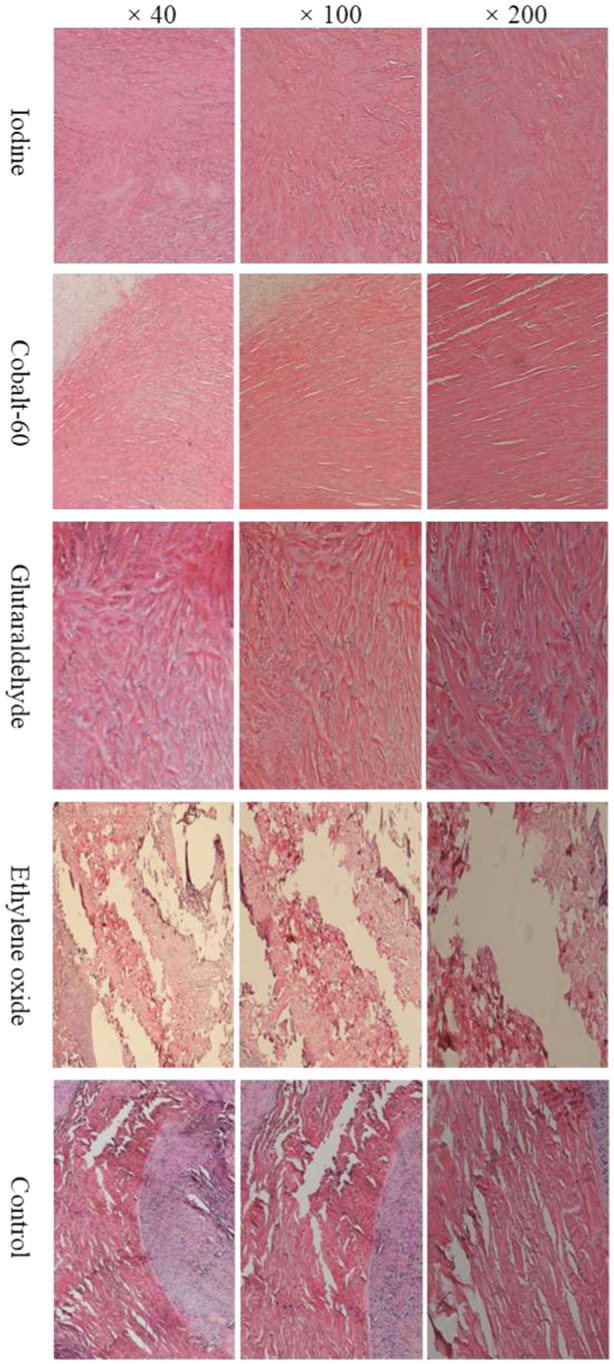
Histological features of the meniscal allograft after 6 weeks of transplantation.

The histological features of the meniscal allografts after transplantation were assessed using H&E staining and were visualized under a light microscope. As illustrated in Figure 4, the meniscal allografts in the iodine group presented the complete structure, with a regular overall arrangement, while regionally distributed small and narrow shuttle‐shaped pores were also observed, which were arranged irregularly. Implanted tissues were found in a few regions in meniscal allografts in the iodine group. The meniscal allografts in the Cobalt‐60 group presented a relatively complete structure with almost regular arrangement and widely distributed spindle‐shaped small pores and small cracks. Implanted tissues were observed in some local regions in meniscal allografts in the Cobalt‐60 group. The structures of meniscal allografts in the glutaraldehyde group were relatively complete, accompanied by regular arrangement and widely distributed small tortuous pores. A great number of inflammatory cells were infiltrated in the implanted tissues in the meniscal allografts in the glutaraldehyde group. The structures of the meniscal allografts in the control group were severely damaged, accompanied by tortuous arrangement and widely distributed large spindle‐shaped cracks. Implanted tissues with a large number of inflammatory cells were observed. The structural damage of the meniscuses in the ethylene oxide group was the most severe among the five groups. A remarkable number of fragmented tissues and large cracks were widely distributed in the meniscal allografts in the ethylene oxide group.

## DISCUSSION

In the present study, an evaluation was conducted to assess both the short‐ and long‐term effects of four common sterilization technologies on disinfecting meniscal allografts. The investigation revealed that iodine, Cobalt‐60, glutaraldehyde and ethylene oxide proved to be effective sterilization techniques for MAT. Notably, iodine demonstrated superior preservation of meniscal allografts in terms of biomechanics and histological structure, while ethylene oxide exhibited the most pronounced detrimental effect on meniscal allograft integrity.

Despite advancements in meniscus‐preserving technologies over the past decades, a recent study highlighted that 65% of arthroscopically identified meniscal tears were irreparable [10]. Surgical interventions for meniscal injuries were considered necessary in the majority of cases, with approximately 1,000,000 meniscectomies performed annually [30]. However, it is acknowledged that meniscus resection surgeries can impact knee joint biomechanics, potentially leading to the early onset of knee joint osteoarthritis. MAT can be a viable treatment option for patients with meniscal deficiencies, aiming to restore biomechanical properties. Sterilization is a crucial step in the preparation of meniscal allografts, as allograft‐associated infections pose significant challenges and are considered the most concerning complication of transplantation [15]. In the present study, the efficacy and long‐term effects of four disinfectants on meniscal allografts in rabbits were compared.

*S. aureus* is one of the most common pathogens for allograft‐associated infections [8, 13, 18, 29], and it is also an opportunistic pathogen with a high level of antibiotic resistance [2], motivating us to select *S. aureus* as bacterial load in the present study. In this study, the differences in *S. aureus* colony count on the meniscuses before sterilization among the five groups were not statistically significant. After sterilization with iodine, Cobalt‐60, glutaraldehyde and ethylene oxide separately, the *S. aureus* colony count in each group was less than 1 CFU/allograft (disinfection rate, 100%), while the *S. aureus* colony count was 415 CFU/allograft in the control group. These results indicated that iodine, Cobalt‐60, glutaraldehyde and ethylene oxide were sufficient to eliminate *S. aureus* that seeded onto the meniscuses. H&E staining was utilized to evaluate the histological features of the meniscuses. It was found that iodine demonstrated optimal preservation in terms of both the biomechanics and structural integrity of the meniscuses, whereas ethylene oxide caused the most severe deterioration of the meniscal tissue.

Despite favourable mid‐term clinical outcomes reported for MAT, the long‐term clinical implications necessitate further investigation [24]. McCormick et al. studied 172 MAT cases, noting a 2‐year survival rate of meniscal grafts at 95% [17]. Saltzman et al. reported survival rates of 98% at 1.7 years, 84% at 5 years and 45% at 10 years after MAT for meniscal grafts [21]. The factors influencing the survival rate of meniscal grafts were complicated. Recipients' age and physical conditions have been found as the most relevant factors in the clinical outcome of MAT [6]. The quality of the meniscal grafts also plays a crucial role in determining the prognosis of transplantation [12]. Previous studies have demonstrated that sterilization could affect the biomechanical and histological properties of meniscal grafts [24]. Therefore, the long‐term effects of four common sterilization agents on the meniscal allografts in rabbits were assessed in the present study.

The meniscus, crucial for weight‐bearing, has been extensively investigated in previous research [32]. Alterations in contact stress have attracted researchers' attention in the context of meniscectomy [32]. Prior research reported a substantial increase in contact pressure (up to 110%) and a reduction in the contact area of the knee joint (up to 75%) in patients with partial‐to‐total meniscectomy, particularly in cadaver studies [4]. Abnormal load conditions in the knee joint can lead to cartilage injury, resulting in vascular invasion, dehydration and ossification of the cartilage. Consequently, meniscectomy is commonly associated with an elevated risk of early‐onset osteoarthritis [32]. MAT has been proposed as an operative option for total or subtotal meniscectomies to restore the native biomechanical properties of the knee joint [32]. Therefore, tests were conducted to assess the effects of four sterilization agents on the biomechanics of meniscal allografts. The results indicated that meniscal grafts in the iodine group endured the highest average load (270.71 ± 62.81 N), while those in the ethylene oxide group experienced only 40.00 ± 22.73 N. The average maximum load in the Cobalt‐60 and glutaraldehyde groups was 182.14 ± 71.29 and 254.29 ± 31.55 N, respectively. Additionally, histological evaluations at six weeks after MAT revealed that the meniscal grafts in the iodine group were well‐preserved, whereas those in the ethylene oxide group exhibited the most severe destruction. These findings suggest that iodine is an efficient and safe disinfectant for meniscal graft preparation, while ethylene oxide is inappropriate for the sterilization of meniscuses.

Iodine has a long history of application in the sterilization of mucosa and skin [34]. Particularly effective for local disinfection therapy, iodine is widely utilized for surface decontamination and exhibits a broad antimicrobial spectrum, targeting bacteria, viruses, fungi and protozoans [34]. Notably, iodine ensures rapid disinfection within seconds, with negligible resistance and minimal allergic or toxic risks [34]. In clinical settings, iodine finds extensive use for procedures, such as skin or mucosa disinfection before or after surgery [34]. Zhao et al. reported that a polyvinylpyrrolidone‐iodine solution could preserve the mechanical properties of the osteogenic allografts [34]. Consistent with the findings of a previous study [34], the present study revealed iodine as the most effective disinfectant for preserving the biomechanical and histological properties of meniscal grafts.

Gamma irradiation was noted to be associated with negative influences on the biomechanical features of the meniscal tissue [9]. The present study, in line with prior research [9], indicated that Cobalt‐60 is inferior to iodine in preserving the biomechanical and histological properties of meniscal grafts. Widely employed as a biocide in clinical settings, glutaraldehyde is also utilized for allograft disinfection [16]. It is also used in allograft disinfection [1]. It has been reported that glutaraldehyde could desensitize the vascular allograft [11]. Powers et al. found that glutaraldehyde‐cross‐linked meniscuses presented an acceptable histocompatibility [19]. The findings of the present study suggested that while glutaraldehyde relatively well preserved the biomechanical and histological properties of meniscal grafts, it was still inferior to iodine in terms of histological preservation. The use of *S. aureus* as a model for assessing sterilization effectiveness in this study could be justified for several reasons. First, *S. aureus* is a common pathogen associated with allograft infections, making it clinically relevant in the context of MAT. Its prevalence in such infections highlights the importance of evaluating sterilization techniques against a representative and opportunistic pathogen. Additionally, *S. aureus* exhibits a high level of antibiotic resistance, posing a significant risk for post‐transplant infections. By using this bacterium, the study could provide a reliable assessment of the sterilization methods' effectiveness, as they must demonstrate the ability to eliminate resistant strains. Furthermore, the significant reduction of *S. aureus* colonies observed after applying the sterilization techniques suggested their efficacy in achieving disinfection, reinforcing the choice of this model. Future studies will expand on this by exploring other pathogens of interest, while the current findings contribute valuable insights into the safety of meniscal allograft procedures.

The strength of this study lies in its novelty as the first investigation to compare the effects of four common sterilization agents on MAT in rabbits. One limitation of the study is that the limit of detection for CFU counts was 1 × 10² CFU/allograft. As a result, any bacterial presence below this threshold could not be quantified, and values below this limit were recorded as ‘<1 CFU/allograft’. This should be taken into account when interpreting the results. The findings highlighted iodine as a potent and safe disinfectant for meniscal grafts. However, it is important to note that this study was conducted at the animal level, and thus, the conclusions drawn from this study require further clinical evaluation.

## CONCLUSIONS

In conclusion, the present research demonstrated that iodine, Cobalt‐60, glutaraldehyde and ethylene oxide could be effective sterilization agents for disinfecting meniscal grafts. Iodine emerged as an efficient and safe disinfectant for MAT, while ethylene oxide posed a significant risk of severe destruction to meniscal allografts.

## AUTHOR CONTRIBUTIONS

**X. S. Wang**: writing—original draft, software, formal analysis. **H. G. Jia**: writing—review and editing, validation, data curation. **D. Q. Gu**: investigation, project administration. **D. Z. Luo**: validation, resources. **Y. T. Zhao**: investigation, data curation. **Z. J. Liu**: resources, supervision. **Y. D. Zhang**: conceptualization, funding acquisition, supervision.

## CONFLICT OF INTEREST STATEMENT

The authors declare no conflicts of interest.

## ETHICS STATEMENT

All animal protocols were approved by the Institutional Animal Care and Use Committee of the Beijing Keyu (KY20230618005). All animal experiments were carried out in accordance with relevant guidelines and regulations. All methods were reported in accordance with ARRIVE guidelines.

## Data Availability

The data that support the findings of this study are available from the corresponding author upon reasonable request.
